# Prognostic significance of serum CA125 in the overall management for patients with gastrointestinal stromal tumors

**DOI:** 10.1186/s12876-023-02655-0

**Published:** 2023-01-26

**Authors:** Chao Sui, Chen Lin, Tingting Tao, Wenxian Guan, Haoran Zhang, Liang Tao, Meng Wang, Feng Wang

**Affiliations:** 1 Medical School of Nanjing University, Nanjing, China; 2 Nanjing Drum Tower Hospital Clinical College of Nanjing University of Chinese Medicine, Nanjing, China; 3 Department of Gastrointestinal Surgery, Nanjing Drum Tower Hospital, The Affiliated Hospital of Nanjing University Medical School, Nanjing, China

**Keywords:** CA125, Gastrointestinal stromal tumor, Progression-free survival, Overall survival, Gastrointestinal tract

## Abstract

**Background:**

Carbohydrate antigen 125 (CA125) is elevated as a tumor marker in many carcinomas, but its association with gastrointestinal stromal tumor (GIST) has received less attention. This study intends to evaluate whether CA125 level can predict tumor progression and overall survival (OS) of GIST patients.

**Methods:**

We retrospectively analyzed the clinical data and follow-up records of GIST patients who underwent surgical resection in Nanjing Drum Tower Hospital from August 2010 to December 2020. All patients were classified according to serum CA125 level. The relationship between CA125 and clinical outcomes was then examined.

**Results:**

A total of 406 GIST patients were enrolled in this study, among which 46 patients had preoperative elevated serum CA125 level and 13 patients with high CA125 level both preoperative and postoperative were observed. Preoperative CA125 concentration was significantly related to rupture status, resection style, tumor site, tumor size, mitotic index, NIH risk grade and c-kit exons. According to Kaplan–Meier curve analysis, high expression of postoperative CA125 was significantly correlated with worse progression-free survival (PFS) and OS among patients with preoperative elevated CA125 level. Ultimately, Cox proportional regression model analysis revealed that increase of preoperative and concurrent postoperative CA125 concentration was an independent predictive factor for PFS.

**Conclusions:**

The concurrent abnormality of serum CA125 before and after operation was an independent risk factor for GIST progression, suggesting its significance as a serum biomarker in the overall management of GIST patients.

## Introduction

The gastrointestinal stromal tumor (GIST) is the most common mesenchymal tumor originating from the gastrointestinal tract [1]. GISTs can occur in any part of the gastrointestinal tract, mostly in stomach (40–50%) and small intestine (20–40%) [2]. The annual incidence of GIST is at least 10–20/1,000,000, with median age being mid 60 s and equal gender distribution [3, 4]. Although the population seems small, we have found that more and more GIST patients diagnosed and treated in clinical practice. Recently, breakthroughs in treatment methods for different malignant potential has been made depends on the progress of molecular biology studies on GIST [5, 6], but complete resection to the primary site with clean margin is still the mainstay and most effective treatment for GIST [7]. In addition, palliative resection is the preferred method for consideration for some metastatic and unresectable GISTs [8, 9]. For those patients underwent surgical resection, tumor recurrence or progression is still an unsolved problem. Researches have shown that 40–50% cases treated with surgery alone relapsed [10]. However, clinical diagnosis of GIST progression mainly depends on imaging examination and lacks effective serum indicators to reflect progression-free survival (PFS) or overall survival (OS).

Serum indicator carbohydrate antigen 125 (CA125) has been confirmed as a tumor marker in many carcinomas, most common in epithelial ovarian tumors with high diagnostic sensitivity and poor specificity [11]. In gastrointestinal cancers, CA125 is also used as an indicator for disease detection and efficacy evaluation. In fact, high serum CA125 level often indicates postoperative recurrence or progression of carcinomas. Many predictive models established in numerous clinical studies described CA125 as an indicator for prognosis and metastasis of gastric cancer [12, 13]. However, the relationship between serum CA125 concentration and GIST progression received few attentions.

Currently, no studies have reported the impact of serum CA125 level on the prognosis of GIST and there is no consensus on whether CA125 should be used as an evaluation index during perioperative period and postoperative review. Therefore, we performed this present research to estimate the effect of serum CA125 level on PFS and OS by collecting preoperative diagnosis and postoperative reexamination data of GIST patients. Our objective is to obtain a simpler and more straightforward indicator to predict tumor progression in advance.

## Methods and materials

### Patient section

This study retrospectively evaluated the medical data of GIST patients who underwent surgical resection in the department of gastrointestinal surgery, Nanjing Drum Tower Hospital from August 2010 to December 2020. The diagnosis of GIST relied on the Chinese and NCCN guidelines. The inclusion criteria were as follows: (1) 18–80 years old; (2) surgical resection; (3) postoperative pathological diagnosis of GIST; (4) detailed and complete medical data. The exclusion criteria were as follows: (1) patients with other carcinomas concomitantly; (2) those with tuberculous peritonitis that may elevate CA125 concentration; (3) patients underwent emergency surgery; (4) those who refused to accept this analysis. At last, a total of 406 patients were enrolled.

### Study design

This research was a single center retrospective examination. The primary outcome was PFS, which was defined as the time from the date of initial surgery to the date of GIST progression or death. Besides, date of last follow-up was the study endpoint for PFS in case of no progression or death. All patients who underwent surgery at our hospital are required to undergo a review in the first month, third month and then every six months after surgery, and among all laboratory tests, CA125 is an important indicator for these patients. The normal range of serum CA125 measured by the Medical Laboratory Department in our center is 0–30.2 U/ml and those who exceed the normal value are defined as having “high serum CA125”. Once the patients had abnormal CA125 during the entire postoperative review, they were defined as having “increased CA125 level both preoperative and postoperative”. And of the 406 enrolled patients, 243 patients with moderate or high NIH risk grade were on TKI after surgery. We first grouped the patients according to the preoperative CA125 level to explore its relationship with clinical outcomes. At the same time, postoperative reexamination provided us the dynamic changes of CA125 in patients after operation. We focused on the patients with preoperative abnormal CA125 concentration in order to find out whether their CA125 level changes in the review were related to GIST progression. Furthermore, laboratory tests, pathological data, gene detection, etc. were used to find other factors affecting the prognosis of GIST.

### Statistical analysis

All relevant statistical analysis were accomplished by using SPSS 25.0 software (IBM Corporation, Armonk, NY, USA) and R software (version 3.5.0). Measurement data were compared by independent-sample *t* test or Mann–Whitney U test while categorical variables using χ^2^ test or Fisher’s exact test. Kaplan–Meier curve analysis was used to estimate PFS and OS, and the differences between the subgroups were assessed by log-rank test. Both univariate and multivariate Cox proportional hazard regression model analysis were used to identify independent factors for GIST recurrence. P value of less than 0.05 was considered to indicate statistical significance.

## Results

### Patient characteristics

Among 406 patients enrolled in this study, 46 patients were found high serum CA125 level before operation. Patients were divided into two groups according to preoperative CA125 level. The clinicopathological parameters between two groups were compared in Table 1, which showed significant difference in rupture status (*P* < 0.001), resection style (*P* = 0.001), tumor site (*P* = 0.025), tumor size (*P* < 0.001), mitotic index (*P* < 0.001), NIH risk grade (*P* < 0.001) and c-kit exons (*P* = 0.001). However, preoperative CA125 level was not associated with clinical characteristics including age, gender, hemorrhage and PDGFRA exons.

**Table 1:** Association between preoperative CA125 level and clinicopathological parameters

	Normal CA125 level (n = 360)	High CA125 level (n = 46)	*P* value
Age^a^	59.00 (51.00,67.00)	57.50 (51.25,68.00)	0.887
Gender (%)			0.056
Male	181 (50.3)	30 (65.2)	
Female	179 (49.7)	16 (34.8)	
Hemorrhage (%)			0.868
Yes	106 (29.4)	13 (28.3)	
No	254 (70.6)	33 (71.7)	
Rupture (%)			**< 0.001**
Yes	1 (0.3)	8 (17.4)	
No	359 (99.7)	38 (82.6)	
Resection style (%)			**0.001**
Complete resection	348 (96.7)	38 (82.6)	
Incomplete resection	12 (3.3)	8 (17.4)	
Tumor site (%)			**0.025**
Stomach	241 (66.9)	23 (50.0)	
Small intestine	103 (28.6)	17 (37.0)	
Colorectum	7 (1.9)	2 (4.3)	
Extra location	9 (2.5)	4 (8.7)	
Tumor size (%)			** < 0.001**
Less than 5 cm	197 (54.7)	5 (10.9)	
5–10 cm	138 (38.3)	19 (41.3)	
More than 10 cm	25 (6.9)	22 (47.8)	
Mitotic index (%)			**< 0.001**
Less than 5/HPF	243 (67.5)	18 (39.1)	
5–10/HPF	55 (15.3)	6 (13.0)	
More than 10/HPF	62 (17.2)	22 (47.8)	
NIH risk grade (%)			** < 0.001**
Extremely low	25 (6.9)	0 (0.0)	
Low	135 (37.5)	3 (6.5)	
Moderate	69 (19.2)	2 (4.3)	
High	131 (36.4)	41 (89.1)	
c-kit exons (%)			**0.001**
Positive	138 (38.3)	29 (63.0)	
Negative	222 (61.7)	17 (37.0)	
PDGFRA exons (%)			0.113
Positive	22 (6.1)	6 (13.0)	
Negative	338 (93.9)	40 (87.0)	

Bold indicates statistically significant (*P* < 0.05)

^a^Median (P25, P75)

### Relationship between preoperative serum CA125 and prognosis

**Fig. 1:**
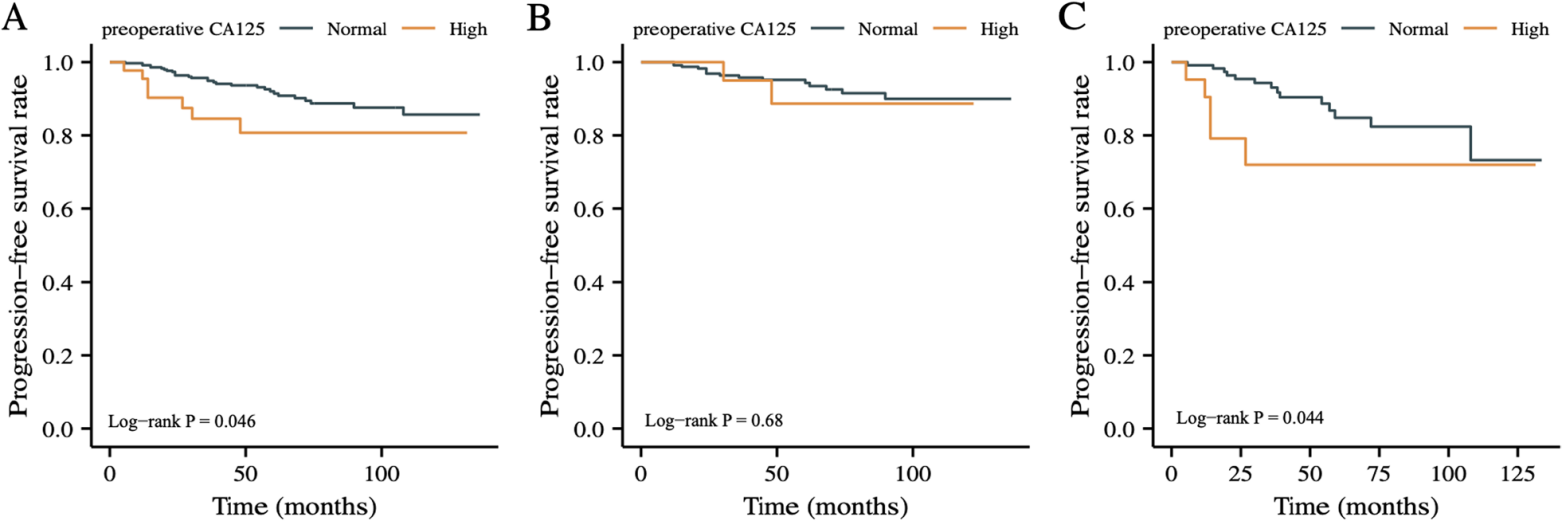
The impact of preoperative serum CA125 level on PFS assessed according to Kaplan–Meier curve analysis. **A** total; **B** patients with GISTs located in stomach; **C** patients with GISTs located in non-stomach

Follow-ups of these 406 patients ranged from 12 to 136 months. At the time of the last follow-up (Dec, 2021), 36 patients were found GIST progression and 42 patients were dead. According to Kaplan–Meier curve analysis, high preoperative serum CA125 level (*P* = 0.046) was related to worse PFS in all included GIST patients (shown in Fig. 1A). Additionally, patients were divided into subgroups based on tumor location. For GISTs located in non-stomach (shown in Fig. 1C), the median PFS of patients with high preoperative CA125 level was significantly lower than patients with normal preoperative CA125 concentration (*P* = 0.044), while no such difference was found in patients with tumors located in stomach (shown in Fig. 1B).

**Fig. 2:**
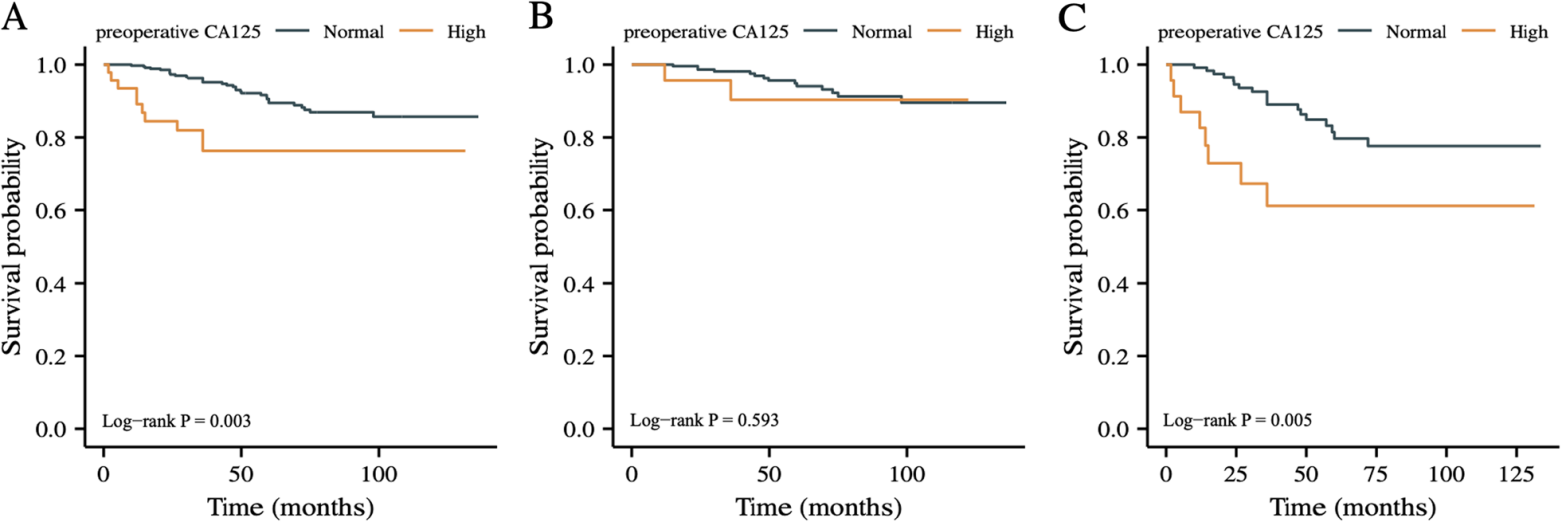
The impact of preoperative serum CA125 level on OS assessed according to Kaplan–Meier curve analysis. **A** Total; **B** patients with GISTs located in stomach; **C** patients with GISTs located in non-stomach

In the subsequent study of the impact of CA125 level on OS, we found that high CA125 level was strikingly relevant to worse OS both in all 406 GIST patients and patients with tumors located in non-stomach (shown in Fig. 2A, C). Whereas, there was no difference in OS between normal and elevated CA125 groups in patients with GISTs located in stomach (shown in Fig. 2B).

### The impact of postoperative serum CA125 level on prognosis

**Fig. 3:**
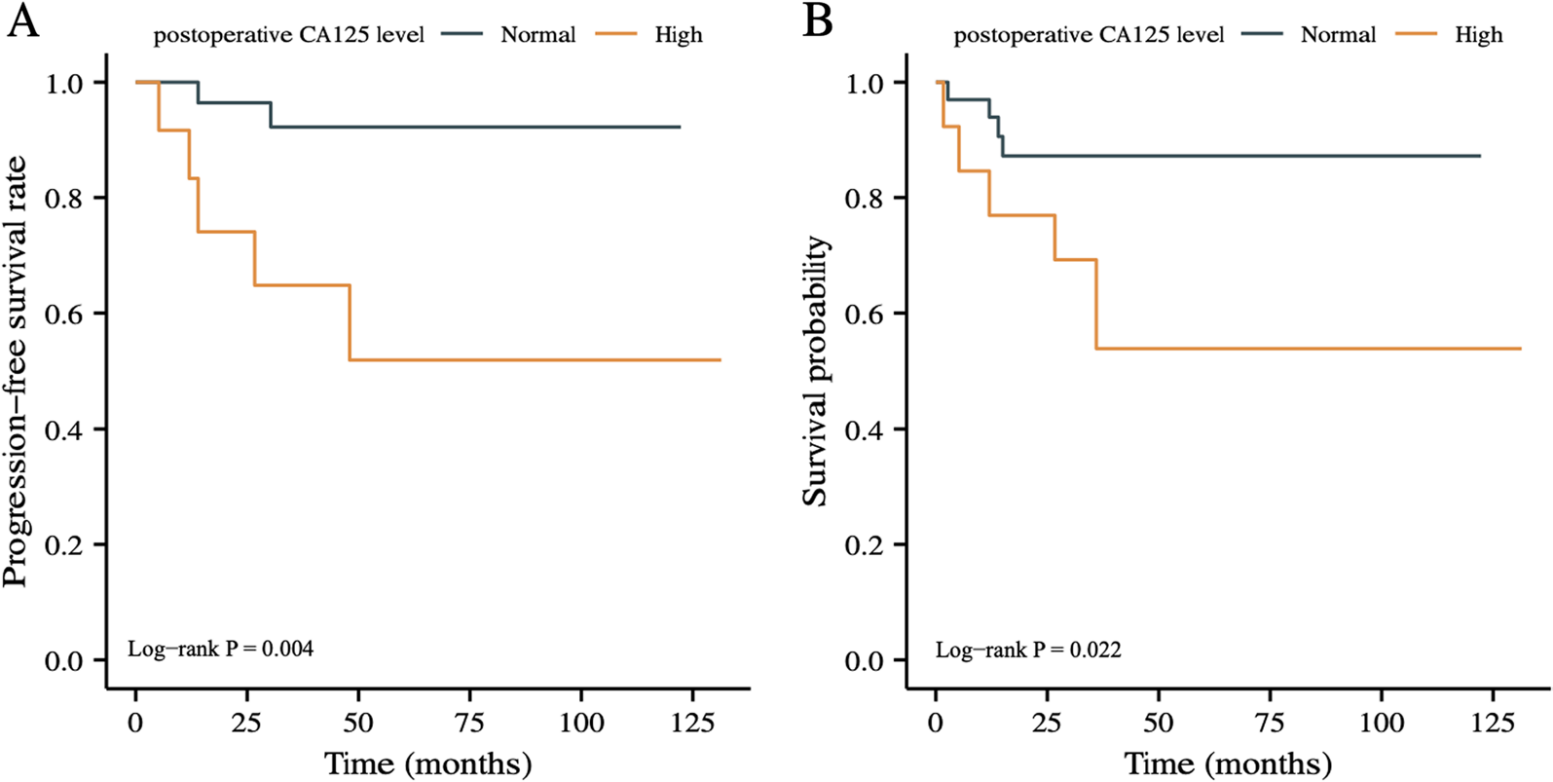
Kaplan–Meier curves of PFS and OS according to postoperative CA125 level among patients with high preoperative serum CA125 level. **A** PFS; **B** OS

To explore deeper relationship between CA125 level and prognosis, we focused on those 46 patients with elevated CA125 level preoperatively and collected laboratory data during their postoperative review. At last, 13 of them were found that serum CA125 level remained elevated. By Kaplan–Meier curve analysis, Fig. 3 presented us that high CA125 level postoperatively was significantly correlated with worse PFS (*P* = 0.004) and OS (*P* = 0.022) compared with those who returned to and maintained normal after operation.

We regarded whether patients have preoperative and concurrent postoperative high CA125 level as a variable in Cox proportional hazard regression model. Univariate analysis showed that resection style (*P* < 0.001, HR = 8.253, 95%CI 3.561–19.044), tumor site (*P* = 0.006, HR = 2.516, 95%CI 1.305–4.849), mitotic index (*P* < 0.001, HR = 3.820, 95%CI 1.930–7.561), NIH risk grade (*P* = 0.001, HR = 4.412, 95%CI 1.831–10.630), preoperative and concurrent postoperative high CA125 level (*P* < 0.001, HR = 6.616, 95%CI 2.571–17.024) were significantly associated with PFS (shown in Table 2). Subsequently, multivariate analysis demonstrated that resection style (*P* < 0.001, HR = 5.536, 95%CI 2.298–13.339), mitotic index (*P* = 0.005, HR = 2.833, 95%CI 1.381–5.811), preoperative and concurrent postoperative high CA125 level (*P* < 0.001, HR = 5.884, 95%CI 2.257–15.341) were independent predictive factors for PFS (shown in Table 2).

**Table 2:** Cox proportional-hazard regression model analysis for PFS

Factors	Univariate analysis PFS		Multivariate analysis PFS	
	HR (95%CI)	*P* value	HR (95%CI)	*P* value
Age				
< 65				
≥ 65	0.586 (0.257–1.339)	0.205		
Gender				
Male				
Female	0.750 (0.387–1.456)	0.395		
Hemorrhage				
No				
Yes	1.307 (0.662–2.579)	0.441		
Rupture				
No				
Yes	1.157 (0.158–8.458)	0.886		
Resection style				
Complete resection				
Incomplete resection	8.253 (3.561–19.044)	< 0.001	5.536 (2.298–13.339)	< 0.001
Tumor site				
Stomach				
Non-stomach	2.516 (1.305–4.849)	0.006	1.599 (0.788–3.243)	0.194
Tumor size				
< 5 cm				
≥ 5 cm	1.937 (0.986–3.803)	0.055		
Mitotic index				
< 5/HPF				
≥ 5/HPF	3.820 (1.930–7.561)	< 0.001	2.833 (1.381–5.811)	0.005
NIH risk grade				
Extremely low or low				
Moderate or high	4.412 (1.831–10.630)	0.001	1.954 (0.658–5.806)	0.228
c-kit exons				
Negative				
Positive	1.901 (0.978 –3.696)	0.058		
PDGFRA exons				
Negative				
Positive	0.739 (0.178–3.079)	0.678		
Preoperative CA125 level				
Normal				
High	2.268 (0.992–5.183)	0.052	0.334 (0.076–1.475)	0.148
Increased CA125 level both preoperative and postoperative				
No				
Yes	6.616 (2.571–17.024)	< 0.001	5.884 (2.257–15.341)	< 0.001

Additionally, Table 3 presented us that age (*P* = 0.047, HR = 1.861, 95%CI 1.009–3.432), resection style (*P* < 0.001, HR = 9.217, 95%CI 4.471–19.003), tumor site (*P* < 0.001, HR = 3.532, 95%CI 1.893–6.589), tumor size (*P* = 0.001, HR = 3.393, 95%CI 1.702–6.764), mitotic index (*P* = 0.012, HR = 2.168, 95%CI 1.183–3.974), NIH risk grade (*P* < 0.001, HR = 6.220, 95%CI 2.441–15.847), preoperative CA125 level (*P* = 0.004, HR = 2.812, 95%CI 1.382–5.723), preoperative and concurrent postoperative high CA125 level (*P* < 0.001, HR = 5.560, 95%CI 2.341–13.204) were proved to be significantly correlated with OS by univariate analysis. In the multivariate analysis, age (*P* = 0.010, HR = 2.286, 95%CI 1.215–4.303), resection style (*P* < 0.001, HR = 5.546, 95%CI 2.558–12.024), tumor site (*P* = 0.026, HR = 2.115, 95%CI 1.092–4.096), NIH risk grade (*P* = 0.003, HR = 4.289, 95%CI 1.623–11.332) were independent risk factors for OS (shown in Table 3).

**Table 3:** Cox proportional-hazard regression model analysis for OS

Factors	Univariate analysis OS		Multivariate analysis OS	
	HR (95%CI)	*P* value	HR (95%CI)	*P* value
Age				
< 65				
≥ 65	1.861 (1.009–3.432)	0.047	2.286 (1.215–4.303)	0.010
Gender				
Male				
Female	0.634 (0.340–1.181)	0.151		
Hemorrhage				
No				
Yes	0.938 (0.480–1.832)	0.851		
Rupture				
No				
Yes	1.987 (0.479–8.241)	0.344		
Resection style				
Complete resection				
Incomplete resection	9.217 (4.471–19.003)	< 0.001	5.546 (2.558–12.024)	< 0.001
Tumor site				
Stomach				
Non-stomach	3.532 (1.893–6.589)	< 0.001	2.115 (1.092–4.096)	0.026
Tumor size				
< 5 cm				
≥ 5 cm	3.393 (1.702–6.764)	0.001	0.987 (0.414–2.356)	0.977
Mitotic index				
< 5/HPF				
≥ 5/HPF	2.168 (1.183–3.974)	0.012	0.797 (0.397–1.602)	0.524
NIH risk grade				
Extremely low or low				
Moderate or high	6.220 (2.441–15.847)	< 0.001	4.289 (1.623–11.332)	0.003
c-kit exons				
Negative				
Positive	1.208 (0.650–2.246)	0.550		
PDGFRA exons				
Negative				
Positive	1.355 (0.483–3.797)	0.564		
Preoperative CA125 level				
Normal				
High	2.812 (1.382–5.723)	0.004	0.894 (0.292–2.737)	0.844
Increased CA125 level both preoperative and postoperative				
No				
Yes	5.560 (2.341–13.204)	< 0.001	2.293 (0.931–5.651)	0.071

## Discussion

This study intended to explore the influencing factors of the long-term clinical outcomes in Chinese GIST patients who underwent surgery through integration and analysis of clinical pathological data. We put the emphasis on discussing the exact relationship between tumor marker CA125 and GIST prognosis by combining data of preoperative laboratory tests and postoperative reexamination. As far as we know, this research established a connection between tumor marker CA125 and GIST for the first time.

Serum tumor marker CA125 has been one of the common laboratory tests for most patients with malignant tumors before operation as its concentration is elevated in numerous tumors [14, 15]. Many clinical studies of gynecologic malignancies incorporated CA125 as a serum marker that indicates tumorigenesis and metastasis [16, 17]. As an easily accessible clinical examination, CA125 also played a key role in the diagnosis of gastrointestinal malignant tumors [18, 19]. Studies have shown that CA125 level was a predictive factor of peritoneal metastases in gastric cancer [20, 21] and normalization of CA125 was relevant with better survival [22]. Thus, we assumed that CA125 level has an impact on the prognosis of GIST patients.

As GISTs are mesenchymal tumors with different malignant potentials, no studies focused on the association between CA125 and GIST. In current clinical practice, there exists a risk of tumor progression and recurrence in GIST patients who underwent radical or incomplete surgical resection, while the diagnosis of GIST progression mainly depends on imaging examination and lacks effective serum indicators. In this present study, we took PFS and OS as outcome measures and found that preoperative high CA125 level had a close relationship with worse PFS and OS through Kaplan–Meier curve analysis. Subsequently, we demonstrated that preoperative and concurrent postoperative high CA125 level were independent risk factors for PFS, but similar results were not observed in multivariate analysis of OS. Considering that PFS is a better outcome index for GISTs, our findings still revealed that comprehensive consideration of both preoperative and postoperative CA125 levels is of great significance in predicting the prognosis of GIST patients. For GIST patients with elevated preoperative CA125 level, special attention should be paid to their postoperative CA125 change as it strongly suggests the risk of postoperative tumor progression.

In addition, this study presented us the significance of overall management of GIST patients and provided a novel perspective. For GIST patients with surgical resection, tumor progression is often unpredictable and easily neglected. Therefore, serum CA125 should become an effective predictor and the dynamic changes of CA125 level needs more attention during the whole management of patients.

The study had several limitations. Firstly, this research was a retrospective examination, which means that selection bias cannot be completely avoided. Secondly, it only included a single-center cohort and further external validation is required to demonstrate whether the present results are feasible for other patient cohorts. Thirdly, GIST patients with normal CA125 level preoperatively lacked reexamination of serum tumor markers and the relationship between postoperative CA125 changes and clinical outcomes in this part of patients cannot be clarified. Thus, multi-center prospective studies are necessary to further clarify the relationship between CA125 level and GIST prognosis. Despite these drawbacks, this study still revealed the prognostic significance of serum CA125 in GIST patients and could provide valued suggestions for clinical practice to some extent.

## Conclusion

In conclusion, this study provided evidence for the relationship between serum CA125 level and clinical outcomes of GISTs. The concurrent abnormality of serum CA125 before and after operation was an independent indicator for progression-free survival in GIST patients with surgical resection, suggesting its significance as a serum biomarker in the overall management of GISTs.

## Data Availability

The data are not publicly available due to privacy or ethical restrictions. Access to the data and the calculation method can be obtained from the corresponding author by email (fengwang36@163.com).

## References

[1] Rubin BP, Heinrich MC, Corless CL (2007). Gastrointestinal stromal tumour. Lancet.

[2] Song H, Xiao X, Liu G, Zhou J (2022). Sarcopenia as a novel prognostic factor in the patients of primary localized gastrointestinal stromal tumor. BMC Cancer.

[3] Miettinen M, Lasota J (2013). Gastrointestinal stromal tumors. Gastroenterol Clin N Am.

[4] Søreide K, Sandvik OM, Søreide JA, Giljaca V, Jureckova A, Bulusu VR (2016). Global epidemiology of gastrointestinal stromal tumours (GIST): a systematic review of population-based cohort studies. Cancer Epidemiol.

[5] Cho H, Nishida T, Takahashi T, Masuzawa T, Hirota S (2022). Impact of the KIT/PDGFRA genotype on prognosis in imatinib-naïve Japanese patients with gastrointestinal stromal tumor. Ann Gastroenterol Surg.

[6] Hølmebakk T, Wiedswang AM, Meza-Zepeda LA, Hompland I, Lobmaier IVK, Berner JM (2021). Integrating anatomical, molecular and clinical risk factors in gastrointestinal stromal tumor of the stomach. Ann Surg Oncol.

[7] Badic B, Gancel CH, Thereaux J, Joumond A, Bail JP, Meunier B (2018). Surgical and oncological long term outcomes of gastrointestinal stromal tumors (GIST) resection- retrospective cohort study. Int J Surg.

[8] Rubió-Casadevall J, Martinez-Trufero J, Garcia-Albeniz X, Calabuig S, Lopez-Pousa A, Del Muro JG (2015). Role of surgery in patients with recurrent, metastatic, or unresectable locally advanced gastrointestinal stromal tumors sensitive to imatinib: a retrospective analysis of the Spanish Group for Research on Sarcoma (GEIS). Ann Surg Oncol.

[9] Roland CL, Bednarski BK, Watson K, Torres KE, Cormier JN, Wang WL (2018). Identification of preoperative factors associated with outcomes following surgical management of intra-abdominal recurrent or metastatic GIST following neoadjuvant tyrosine kinase inhibitor therapy. J Surg Oncol.

[10] Joensuu H, Vehtari A, Riihimäki J, Nishida T, Steigen SE, Brabec P (2012). Risk of recurrence of gastrointestinal stromal tumour after surgery: an analysis of pooled population-based cohorts. Lancet Oncol.

[11] Funston G, Hamilton W, Abel G, Crosbie EJ, Rous B, Walter FM (2020). The diagnostic performance of CA125 for the detection of ovarian and non-ovarian cancer in primary care: a population-based cohort study. PLoS Med.

[12] Hu X, Yang Z, Chen S, Xue J, Duan S, Yang L (2021). Development and external validation of a prognostic nomogram for patients with gastric cancer after radical gastrectomy. Ann Transl Med.

[13] Xue B, Jiang J, Chen L, Wu S, Zheng X, Zheng X (2021). Development and validation of a radiomics model based on (18)F-FDG PET of primary gastric cancer for predicting peritoneal metastasis. Front Oncol.

[14] Li Z, Zhao J (2021). Clinical efficacy and safety of crizotinib and alectinib in ALK-positive non-small cell lung cancer treatment and predictive value of CEA and CA125 for treatment efficacy. Am J Transl Res.

[15] Lee J, Kim JM, Lee YH, Chong GO, Hong DG (2022). Correlation between clinical outcomes and serum CA-125 levels after standard treatment for epithelial ovarian cancer. Anticancer Res.

[16] Behnamfar F, Esmaeilian F, Adibi A, Rouholamin S (2022). Comparison of ultrasound and tumor marker CA125 in diagnosis of adnexal mass malignancies. Adv Biomed Res.

[17] Ran C, Sun J, Qu Y, Long N (2021). Clinical value of MRI, serum SCCA, and CA125 levels in the diagnosis of lymph node metastasis and para-uterine infiltration in cervical cancer. World J Surg Oncol.

[18] Guleken Z, Bulut H, Gültekin G, Arıkan S, Yaylım İ, Hakan MT (2021). Assessment of structural protein expression by FTIR and biochemical assays as biomarkers of metabolites response in gastric and colon cancer. Talanta.

[19] Wang Z, Mo TM, Tian L, Chen JQ (2021). Gastrin-17 combined with CEA, CA12-5 and CA19-9 improves the sensitivity for the diagnosis of gastric cancer. Int J Gen Med.

[20] Yang C, Yang Y, Huang X, Li H, Cheng H, Tong S (2020). A nomogram based on clinicopathologic features and preoperative hematology parameters to predict occult peritoneal metastasis of gastric cancer: a single-center retrospective study. Dis Markers.

[21] Huang J, Chen Y, Zhang Y, Xie J, Liang Y, Yuan W (2022). Comparison of clinical-computed tomography model with 2D and 3D radiomics models to predict occult peritoneal metastases in advanced gastric cancer. Abdom Radiol (NY).

[22] Hu C, Zhang Y, Xu J, Chen W, Yu P, Wang Y (2022). Prognostic significance of serum tumor marker normalization in the perioperative period for patients with advanced gastric cancer. Ann Transl Med.

