# Correction to: Prognostic significance of serum CA125 in the overall management for patients with gastrointestinal stromal tumors

**DOI:** 10.1186/s12876-023-02686-7

**Published:** 2023-03-30

**Authors:** Chao Sui, Chen Lin, Tingting Tao, Wenxian Guan, Haoran Zhang, Liang Tao, Meng Wang, Feng Wang

**Affiliations:** 1 Nanjing Drum Tower Hospital, The Affiliated Hospital of Nanjing University Medical School, Nanjing, China; 2 Department of Gastrointestinal Surgery, Nanjing Drum Tower Hospital, The Affiliated Hospital of Nanjing University Medical School, Nanjing, China; 3 Nanjing Drum Tower Hospital Clinical College of Nanjing University of Chinese Medicine, Nanjing, China

**BMC Gastroenterology (2023) 23:25**

10.1186/s12876-023-02655-0

After publication of this article [1], the authors reported that the details for affiliation 1 were incorrectly given as ‘Medical School of Nanjing University, Nanjing, China’, but should have been ‘Nanjing Drum Tower Hospital, The Affiliated Hospital of Nanjing University Medical School, Nanjing, China’.

The original article [1] has been corrected.
